# Acrylamide Determination in Infant Formulas: A New Extraction Method

**DOI:** 10.3390/molecules30244718

**Published:** 2025-12-09

**Authors:** Sumeyra Sevim, Rosalia Lopez-Ruiz, Antonia Garrido-Frenich

**Affiliations:** 1 Department of Nutrition and Dietetics, Faculty of Health Sciences, Ankara Medipol University, 06500 Ankara, Türkiye; sumeyra.sevim@ankaramedipol.com; 2 Research Group “Analytical Chemistry of Contaminants”, Department of Chemistry and Physics, Research Centre for Mediterranean Intensive Agrosystems and Agri-Food Biotechnology (CIAIMBITAL), University of Almeria, Agrifood Campus of International Excellence, ceiA3, E-04120 Almeria, Spain; agarrido@ual.es

**Keywords:** acrylamide, infant formula, LC, sample preparation, MS, milk

## Abstract

Infant formulas are specialized foods designed for babies and toddlers who cannot be exclusively breastfed. However, acrylamide (AA) may form during the thermal processing involved in their production. Although chromatographic techniques offer high sensitivity and detection capability for AA analysis, their application remains limited due to the complexity of diverse food matrices, high operating costs, time requirements, and environmental concerns. A new validated liquid chromatography–mass spectrometry (LC-MS) protocol for AA detection in infant formula was developed using sequential hydration, acetonitrile (ACN) precipitation, and dual-sorbent clean-up, which minimized matrix effects and ensured clarity and high reproducibility. The validated method demonstrated excellent linearity (R^2^ = 0.9985, solvent-based; 0.9903, matrix-based), a pronounced matrix effect (−67%), satisfactory sensitivity (limit of detection, LOD: 10 µg/kg; limit of quantification, LOQ: 20 µg/kg), and consistent recovery (82–99%) with less than 15% variation. AA analysis was performed on 31 infant formula samples. The highest individual AA level (268.2 µg/kg) was detected in an amino acid-based formula intended for infants under one year of age while the highest mean concentration was found in cereal-based samples (188.1 ± 100.8 µg/kg), followed by goat’s milk-based (52.7 ± 25.67), plant-based (48.8 ± 31.68), and cow’s milk-based (27.5 ± 29.62) formulas (*p* < 0.001). The wide variability in AA concentrations among infant formulas can be attributed to differences in formulation, ingredient composition, manufacturing processes, and analytical methodologies. These findings highlight the need for continuous monitoring of AA levels in infant foods to ensure their safety.

## 1. Introduction

Commercially available infant formulas used as partial or complete substitutes for breast milk are consumed worldwide to feed infants and young children aged 0–36 months [1]. There are two main forms of infant formula: powdered, which must be reconstituted with water, and ready-to-feed liquid. Among these, powdered formula is the most widely available, shelf-stable, and commercially distributed type [2]. As infant formulas play a critical role during the first months of life, they must be free from potentially hazardous toxic compounds from a food safety perspective [3].

Infant formulas are manufactured using a wide range of processing techniques, incorporating both thermal and non-thermal technologies, which result in distinct final product compositions [2]. Conventional thermal processes are essential for producing powdered products and for achieving pasteurization or sterilization effects. These methods effectively reduce microbial loads and extend product shelf life; however, maintaining an appropriate balance between microbial safety and the prevention of undesirable chemical changes is crucial [4,5]. Moreover, thermal treatments have a pronounced impact on the physicochemical properties of infant formulas due to complex interactions among proteins, carbohydrates, and lipids. During production, the Maillard reaction can occur when protein- and sugar-containing ingredients are exposed to heat, leading to the formation of harmful compounds such as acrylamide (AA) [6].

AA is an α,β-unsaturated carbonyl compound formed in certain foods processed at temperatures above 120 °C through the reaction between amino acids and reducing sugars such as glucose and fructose [7]. According to the International Agency for Research on Cancer (IARC), AA is classified as a Group 2A compound, meaning it is “probably carcinogenic to humans” [8]. Over the past two decades, studies have shown that dietary exposure to AA can cause carcinogenic, neurotoxic, genotoxic, and reproductive or developmental effects in both humans and animals [9,10,11,12]. Reflecting its recognized carcinogenic potential, AA was listed among the high-priority agents for re-evaluation in the IARC Monographs Programme for 2020–2024 [13].

AA in food poses a particularly high risk to infants and young children due to their specific physiological characteristics, including higher dietary intake per kilogram of body weight, elevated resting metabolic and respiratory rates, and a larger body surface area relative to adults [3]. The European Commission has established “indicative values” for AA in various food categories based on monitoring data from Member States between 2007 and 2011. Although these values are not safety thresholds, exceeding them requires manufacturers to take corrective measures to limit AA formation. In the baby food category, reference levels of 50 µg/kg for non-cereal-based products and 80 µg/kg for foods containing plums have been set [14]. A recent meta-analysis reported that the mean AA content in baby foods (156.3 µg/kg) exceeded the reference limits (40–150 µg/kg), which is concerning given children’s greater susceptibility compared to adults [15]. “Baby foods other than processed cereal-based” were identified as the largest contributors to infants’ total AA exposure according to European data, with infants estimated to ingest three times more AA per day than adults [16].

In recent years, liquid and gas chromatographic techniques (LC-MS/MS and GC-MS) have become the standard analytical approaches for AA determination due to their high sensitivity and detection capability. Several extraction and clean-up methods, such as solid–liquid extraction (SLE), solid-phase extraction (SPE), and QuEChERS, have been employed to isolate AA from food matrices. However, the application of these techniques remains limited by the complexity of food matrices, high operational costs, time-consuming procedures, and environmental impact [9,17,18,19]. Therefore, there is a growing need for simple, robust, and broadly applicable analytical methods compatible with widely available detection systems. To address these limitations, this study proposes a simplified extraction strategy designed to reduce sample-handling steps and matrix interferences, particularly for powered infant formula matrices. In this context, the objective of the study was to establish a sensitive and selective analytical approach for AA determination in powdered infant formulas. For this purpose, an optimized SLE-based extraction combined with a dispersive solid-phase extraction (d-SPE) clean-up and liquid chromatography–triple quadrupole mass spectrometry (LC-QqQ-MS) detection was proposed.

## 2. Results and Discussion

### 2.1. Optimization and Validation of the Extraction and LC-MS Method

The MS characterization and chromatographic separation were previously optimized following the procedure described by López-Ruiz et al. [18]. The extraction procedure was further refined based on a previously developed method that employed SLE with water and a clean-up step using C18 and Primary Secondary Amine (PSA) sorbents. In that protocol, an infant formula sample (1 g) was hydrated with 5 mL of water and directly subjected to purification. However, this approach exhibited significant drawbacks when applied to powdered infant formula, as reliable AA detection could not be achieved under these conditions. Pronounced matrix effects were observed, mainly due to the high protein and lipid content of the matrix. Moreover, filtration of the extracts was problematic, as they could not be readily prepared for LC-MS injection.

To address these issues, several extraction solvents and mixtures such as dichloroethane, ACN/water/formic acid, and ACN/water were tested. None of these conditions yielded satisfactory results: AA was either undetectable or exhibited inconsistent recoveries (below 45%). These unsuccessful attempts highlighted the challenge of efficiently extracting AA from infant formulas without incorporating an effective protein precipitation step. To overcome this limitation, ACN was introduced following an initial hydration phase with water. This modification improved protein precipitation, reduced matrix complexity, and enhanced AA recovery to 85%.

For method validation, the parameters established in the SANTE/11312/2021 guidelines [20], were evaluated, including linearity, matrix effect (ME), limits of quantification (LOQ) and detection (LOD), as well as trueness and precision (intra- and inter-day).

Linearity and matrix effects were assessed using calibration curves prepared both in solvent (ACN/water) and in matrix-matched extracts at concentrations of 20 (LOQ), 50, 100, 200, 300, 400 and 600 µg/kg. Relative peak areas were calculated as the ratio of the AA peak area to that of the internal standard (AA-d_3_). The determination coefficients (R^2^) demonstrated excellent linearity, with values of 0.9985 for the solvent-based calibration curve and 0.9903 for the matrix-based curve. The matrix effect, calculated according to Equation (1), indicated strong signal suppression for AA (−67%). Considering the strong matrix effect observed, AA concentration in all samples were quantified using the matrix- based curves to ensure accurate quantification.

ME (%) = ((slope in matrix)/(slope in solvent) − 1)∙100(1)

The LOQ was defined as the lowest concentration providing acceptable recovery (70–120%) and precision (RSD < 20%), which was established at 20 µg/kg for AA. The LOD, determined as the lowest concentration yielding a signal-to-noise (S/N) ratio below 3, was set at 10 µg/kg.

Trueness, expressed as recovery (%), and precision, expressed as relative standard deviation (RSD), were evaluated at two concentration levels and 5 replicates each level: One sample of each type of main ingredient (as representative groups) were prepared according to Section 3.3, were spiked with the analytical standard of AA at the LOQ (20 µg/kg) and 250 µg/kg. Recovery rates were 82% at the LOQ and 99% at 250 µg/kg, while intra- (same day) and inter-day (different days *n* = 4) precision values remained below 15% at both levels.

### 2.2. Samples Analysis

The analyzed infant formula samples were classified according to age group, main ingredient, and formula type to ensure representativeness across different product categories, and this distribution is shown in Table 1. Detailed information regarding the 31 infant formula samples analyzed, including age category, main ingredient (e.g., cow’s milk-based), formula type, brands codes (B1–12), primary protein and carbohydrate source and the AA concentration found in each sample, is provided in Supplementary Table S1. Among the analyzed products, most (*n* = 14; 45.2%) were intended for infants aged 0–6 months, followed by formulas for infants < 1 year (*n* = 8; 25.8%), >6 months (*n* = 3; 9.7%), >1 year (*n* = 3; 9.7%), 0–36 months (*n* = 2; 6.5%), and 6–12 months (*n* = 1; 3.2%). Based on the main ingredient, cow’s milk-based formulas represented the majority (*n* = 21; 67.7%), followed by goat’s milk-based (*n* = 5; 16.1%), plant-based (*n* = 2; 6.5%), cereal-based (*n* = 2; 6.5%), and amino-acid based (*n* = 1; 3.2%) formulations. In terms of functional classification, products were categorized as either standard formulas (*n* = 18; 58.1%) or therapeutic/special formulas (*n* = 13; 41.9%) according to product labeling.

Table 2 structure allows comparison of AA concentration levels across different brands. The measured AA concentrations in the analyzed infant formulas showed substantial variability across age groups and main ingredients (Table 2). The highest mean AA concentration was observed in the amino-acid-based formula of brand B1 intended for infants under one year of age (mean = 268.2 µg/kg), followed by the same brand’s cereal-based formula for infants older than six months (mean = 259.4 µg/kg) and the cereal-based therapeutic formula of brand B4 for infants aged 0–36 months (mean = 116.8 µg/kg). In contrast, AA levels were considerably lower in cow’s milk- and plant-based formulas produced by the same brand (B1) for different age groups.

This variability may be attributed to differences in carbohydrate sources used during formulation. Specifically, the primary carbohydrate in the high-AA products was glucose syrup that was a reducing sugar involved in the formation of Schiff bases asparagine (Asn) in the Maillard reaction to produce AA [19]. Thus, these products were more prone to AA formation than other infant formulas. Moreover, protein source of the products was free amino acid. In comparison, other formulas from the same brand primarily contain lactose as the carbohydrate source, which generates significantly less AA under similar processing conditions. Interestingly, despite also containing glucose syrup, the formula from brand B7 exhibited relatively low AA levels (25.8 µg/kg). This situation is probably also related to the fact that it contains milk protein rather than free amino acids as a protein source.

These results highlight the critical role of formulation design and ingredient selection in influencing AA levels in infant formulas, even among products from the same manufacturer.

**Figure 1:**
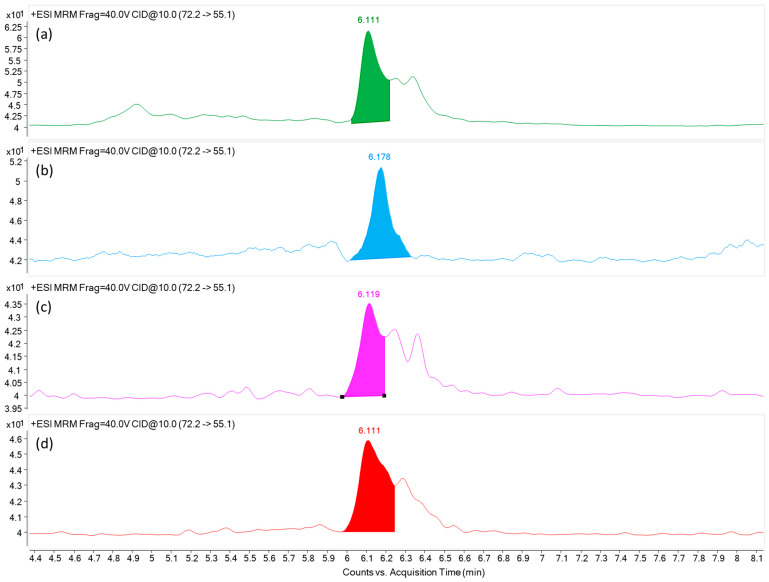
Extracted ion chromatogram of AA in infant formula samples. (**a**) goat milk (Sample 12); (**b**) oatmeal (Sample 24); (**c**) soybean (Sample 23); (**d**) Rice (Sample 30).

Overall, the mean AA concentration varied statistically significantly between formula groups, according to a one-way ANOVA (*p* < 0.001), are presented in Supplementary Table S2. Tukey’s post-hoc test, excluding the group with a single observation (amino acid-based), revealed that the AA levels in cereal-based formulas were considerably higher than those in cow’s milk-based formulas, goat’s milk-based formulas, and plant-based formulas (*p* < 0.001) while there were no significant differences among the other groups (*p* > 0.05). Accordingly, cow’s milk-based formulas showed the lowest average AA levels (0–77.5 µg/kg; mean = 27.5 ± 29.6 µg/kg), while goat milk-based formulas showed approximately double the concentration (23.7–80.0 µg/kg; mean = 52.7 ± 25.7 µg/kg), but there was no statistically significant difference. Plant-based formulas displayed moderate AA levels (26.4–71.2 µg/kg; mean = 48.8 ± 31.7 µg/kg), whereas cereal-based formulas contained significantly higher concentrations (116.8–259.4 µg/kg; mean = 188.1 ± 100.8 µg/kg) compared to the other groups (*p* < 0.001). Figure 1 shows an extracted ion chromatogram (EIC) for AA detected in infant formulas derived from different main ingredients.

According to the European Food Safety Authority [14], AA concentrations in baby foods and infant formulas vary significantly depending on the product type and ingredients. The mean AA concentration reported in non-cereal-based infant formulas was 24 µg/kg. Notably, formulas containing prunes showed much higher mean levels (101 µg/kg) compared with those without prunes (20 µg/kg), primarily due to the elevated sugar and free asparagine content in dried fruits, which are key precursors for AA formation.

AA concentrations in infant formulas vary widely worldwide due to differences in formulation and composition, production and processing parameters, and analytical detection methods [3]. A study conducted in Iran reported extremely high AA levels in infant formulas, ranging from 48 to 5835 µg/kg. The authors concluded that among all factors, protein composition had the greatest impact on AA formation in powdered formulas, while both formulation and ingredient composition also showed significant effects [17]. Similar findings were observed in infant formula powders analyzed in Colombia [21]. The results of this study also support these findings. In the two samples with the highest AA content, the protein source consists of free amino acids and the carbohydrate source consists of glucose syrup. The presence of free amino acids and reducing sugars, which increase acrylamide formation in the Maillard reaction, explains the high AA levels observed in these products.

Conversely, French “Follow-on formula” and “Infant formula” products contained relatively low AA levels (0.14/2.2 µg/kg for the lower-bound assumption and 0.60/2.9 µg/kg for the upper-bound assumption) compared with previous studies [22]. More recently, a study conducted in Türkiye reported AA levels ranging from <LOQ to 578 µg/kg, with a mean of 69.9 µg/kg. The mean AA concentrations were 45.1 µg/kg for 0–6 months, 62.5 µg/kg for 7–12 months, and 88.9 µg/kg for >12 months. The highest level (251 µg/kg) was detected in a brand 4 formula intended for children > 12 months [23].

### 2.3. Comparison of the Method Used with Alternative Techniques for Determination of AA in Infant Formula

Recent advances in analytical methodologies—such as simplified derivatization, SPE, and advanced purification techniques—have substantially improved detection limits and analytical reliability [9]. SPE, using either normal-phase or reversed-phase sorbents, enables further refinement of extracts [24]. This sample preparation technique is widely used in analytical chemistry to concentrate and isolate target compounds from complex matrices prior to analysis. The solid-phase sorbent material selectively retains analytes while removing unwanted matrix components [25]. Due to its versatility and adaptability, SPE is applicable to a broad range of samples, including biological, food, and environmental matrices [26].

Accordingly, the newly developed method in this study incorporates an SPE-based extraction step specifically optimized for infant formula matrices to achieve enhanced sensitivity and reliability. The method demonstrated excellent analytical performance, with a linear range of 20–600 µg/kg (R^2^ = 0.9905), LOD and LOQ values of 10 μg/kg and 20 μg/kg, respectively, and recovery rates ranging from 70% to 120%. The method precision (RSD) was below 15% across all concentration levels, confirming satisfactory repeatability. The comparison of alternative techniques for AA detection in infant formulas is summarized in Table 3. Lee et al. [27] employed the isotope dilution–liquid chromatography/mass spectrometry (ID–LC/MS) method to develop and validate a robust analytical procedure aimed at establishing a reliable reference material for accurately quantifying AA in infant formula. The ID-LC/MS approach demonstrated excellent accuracy and reliability, achieving low repeatability (<1.1%) and intermediate precision (<1.4%). The combined use of chloroform–methanol (MeOH) extraction and Oasis PRiME SPE clean-up effectively minimized matrix effects, while the use of the ^13^C_3_-AA internal standard further enhanced quantification accuracy. However, despite its high analytical performance, this method requires costly instrumentation and consumables, and its complexity demands highly trained personnel. These factors limit their routine application in standard analytical laboratories [27]. Basaran and Aydın [23] confirmed that LC-MS/MS is a reliable and accurate technique for AA determination in infant formula. Their validation results and high analytical performance demonstrated that the method is suitable and effective for quantitative analysis of AA in such matrices.

Similarly, Ghiasi et al. [17] developed a dispersive liquid–liquid microextraction-based microextraction (ME)-GC/MS method that exhibited excellent performance for AA determination in powdered infant formulas. This approach enabled rapid and efficient extraction, providing high sensitivity with a low detection limit (0.6 ng/g). The method also showed high selectivity, acceptable accuracy and reproducibility (RSD 2.9%), a strong enrichment factor (140), and improved recovery. Its user-friendly design and the use of low-cost GC analysis enhance its practicality. Additionally, the two-step sample preparation process (hydrolysis followed by microextraction) further improved sensitivity, selectivity, and recovery. Mastovska et al. [28] developed a method allowing rapid and simple sample preparation for AA determination in diverse food matrices, which was later effectively applied to infant formulas by Pacetti et al. [21]. The method proved reliable in terms of accuracy and reproducibility, while offering high sample throughput, low operational cost, reduced contamination risk, and the use of an isotopic internal standard (AA-d_3_). The combination of ACN-based extraction, hexane-assisted oil removal, and MgSO_4_^−^NaCl salt addition ensured efficient phase separation, whereas dispersive d-SPE with PSA sorbent effectively reduced matrix effects and removed interfering compounds, particularly beneficial in LC-MS/MS analysis [28].

Table 3 highlights that the current method efficiently eliminates matrix interactions, reduces solvent consumption and shortens sample preparation time compared to previous methods, emphasizing the performance of the analytical method. However, its analytical sensitivity was comparatively lower than that of several previously reported methods, as reflected by the higher LOQ (20 µg/kg) shown in Table 3. Importantly, these detection limits were obtained using a streamlined, time-efficient SLE followed by d-SPE procedure specifically optimized for infant formula matrices. The combined use of PSA and DSC-18 sorbents during the clean-up stage ensured efficient removal of both polar and non-polar matrix components. PSA effectively eliminated polar substances such as organic acids, pigments, and certain sugars, while C18 retained lipids and hydrophobic co-extractives. This workflow reduced matrix interferences, minimized solvent consumption, and shortens sample preparation time. Therefore, the proposed method provides a reliable and effective substitute with acceptable recovery (82–99%) and precision (RSD < 15%) for determining AA in powdered infant formulas by combining analytical robustness with practical applicability.

**Table 3:** Evaluation of the alternative techniques for detecting AA in baby formulas.

Reference	Extraction	Cleanup	Analytical Instrument	Results	LOD & LOQ
This study	0.75 g sample + 10 μL AA-d_3_ 5 mL water 5 mL ACN	100 mg DSC-18 100 mg PSA	LC-QqQ-MS	0–268.2 μg/kg in infant formulas	10 & 20 µg/kg
[27]	1 g sample + AA-^13^C_3_ 10 mL water 10 mL of a chloroform–MeOH (2:1, *v*:*v*)	Oasis PRiME HLB (6 cc, 200 mg) SPE cartridges Oasis PRiME MCX (6 cc, 150 mg) SPE cartridges	ID-LC/MS	55.7 ± 2.1 μg/kg in infant formulas	0.03 & 0.1 µg/kg
[23]	1 g sample + 1 mL AA-d_3_ 9 mL water 5 mL n-hexane Filtration through a 0.45 μm PVDF	Oasis HLB cartridges A Bond Elut Accucat SPE cartridges	LC-MS/MS	<LOQ—578.0 μg/kg in infant formulas	3.0 & 10.0 µg/kg
[29]	MSPD extraction: 0.50 g sample 2 g C18 20 mL of n-hexane 5 mL water	Bromination: Potassium bromide HBr (48% *w*/*w*) Bromine water (3% *w*/*v*) NaCl Ethyl acetate:n-hexane mixture (4:1 *v*/*v*) Sodium sulfate	GC/MS	<LOD—109 µg/kg in baby food (biscuits, multigrain meal, sweet snacks, savory snacks, baby food with plum puree)	10.0 & 30.0 µg/kg
[17]	1 g sample 3 mL n-hexane 200 µL acetamide 7 mL KOH and ethanol (80:20)	Carrez I and Carrez II (1 mL each) Xanthydrol Hydrochloric acid K_2_HPO_4_ (2 mol/L) and KOH (2 mol/L) d-LLME: Tetrachloroethylene Ethanol	ME-GC/MS	48–5385 µg/kg in infant formulas	0.6 & 1.98 µg/kg
[30]	1 g sample + AA-^13^C_3_ 20 mL of 10 mM formic acid in water	Carrez I and Carrez II (0.5 mL each) Oasis MCX cartridge	LC-MS/MS	<LOD—92.4 μg/kg in infant cereal	3.0 & 10.0 µg/kg
[31]	1 g sample + AA-^13^C_3_ 9 mL water	SPE (with two stages): OASIS SPE cartridge Bond Elut-Accucat SPE cartridge	LC-MS/MS	5–1788 µg/kg in infant formula	10 µg/kg (LOQ)
[22]	2 g sample + AA-d_5_ 8 mL water SLE	Oasis HLB SPE cartridge Amicon Ultra-15 filtration cartridge (Ultra-filtration) Isolute Multimode cartridge (Purification)	LC-MS/MS	2.4–18 µg/kg in infant formula and follow-on formulas	2.0 & 5.0 µg/kg
[21]	QuEChERS/purification ^†^ 2 g sample 5 mL of n-hexane 10 mL of water 10 mL of ACN 4 g of MgSO_4_ + 0.5 g of NaCl	d-SPE ^†^: PSA + MgSO_4_	GC/MS ^†^	<LOQ—1821 µg/kg in infant powdered formula	25.0 & 75.0 µg/kg
[32]	3 g sample + AA-d_3_ 20–40 mL water 10 mL n-hexane	Carrez I and II solutions (1 mL each) SPE clean-up: Bakerbond Carbon column	LC-MS/MS	32–312 µg/kg in follow-on formulas	2.5 μg/kg (LOQ)

^†^ This method was received by Mastovska and Lehotay (2006) [28].

## 3. Materials and Methods

### 3.1. Chemical

AA (99% of purity) and AA-d3 (500 mg/L in ACN) were provided by Sigma-Aldrich (St. Louis, MO, USA). Stock standards of AA were prepared at 10 mg/L and 1 mg/L in high-performance liquid chromatography (HPLC)-grade water and AA-d3 was prepared at 5 mg/L in ACN, working solutions are stored at 4 °C until use.

For AA extraction, ACN, water HPLC grade, PSA SPE bulk packing (52738-U) and DSC-18 SPE bulk packing (52600-U) were provided from Merck (St. Louis, MO, USA). For mobile phase MeOH and formic acid LC-MS grade were obtained from Merck.

### 3.2. Sampling

A total of 31 infant formula samples were obtained from Brazil and Türkiye based on availability and existing research collaborations. The Brazilian samples included in the study were locally produced and belonged to European brands with international distribution whereas the Turkish samples represented locally produced domestic brands. A comprehensive and representative analysis was ensured by including a diverse range of infant formulas, comprising premature formulas, starter formulas, follow-up formulas, anti-reflux formulas, lactose-free formulas, hypoallergenic formulas, specialized formulas, and formulas based on soy, rice, and goat milk (Table 1). All samples were stored in their original packaging at room temperature until the study.

### 3.3. Acrylamide Extraction

The sample quantity and dilution procedure for AA extraction were established according to the manufacturer’s preparation instructions (1 tablespoon (4.65 g) per 30 mL of water). All samples were prepared in triplicate (*n* = 3), 0.75 g of powdered infant formula was weighed into a 15 mL Falcon tube, followed by the addition of 5 mL of water.

Each sample was vortexed for 2 min to ensure complete homogenization. After 5 min, 10 µL of the internal standard solution (AA-d_3_, 5 µg/L in ACN) and 5 mL of ACN were added. The tubes were then placed on a rotary shaker and agitated for 30 min at room temperature. Subsequently, samples were centrifuged at 7500 rpm (≈8160× *g*) for 10 min at 4 °C.

Meanwhile, 100 mg of DSC-18 and 100 mg of PSA sorbents were weighed into a separate 15 mL tube. An aliquot of 1.5 mL of the supernatant was transferred into this tube and vortexed for approximately 1 min to facilitate clean-up. The mixture was then centrifuged again at 7500 rpm (≈8160× *g*) for 10 min at 4 °C. Finally, a 1 mL aliquot of the clarified supernatant was transferred into an LC–MS vial for analysis.

### 3.4. Analysis Conditions

The detection of AA was carried out using a method previously developed by the research group [18] employing an HPLC system, specifically the Agilent 1290 RRLC Infinity I (Agilent Technologies, Pune, MA, USA), equipped with a binary pump (G4220A), an autosampler thermostat (G1330B), and a column compartment thermostat (G1316C). The chromatographic system was hyphenated to an Agilent 6460 A triple quadrupole mass spectrometer, operated with a Jet Stream electrospray ionization (ESI) source (G1958-65138). Chromatographic separation was achieved on an ACE Excel 3 Super C18 analytical column (150 × 4.6 mm, 3.0 μm particle size; Avantor, VWR, Radnor, PA, USA).

The mobile phase consisted of (A) water containing 0.1% formic acid (*v*/*v*) and (B) MeOH. Gradient elution was initiated at 95% A, maintained for 1 min, followed by a linear decrease to 0% A within 1–5 min, held constant for 3 min. Subsequently, the gradient was returned to the initial condition (95% A) in 1 min and maintained for an additional 1.5 min. The flow rate was set at 0.4 mL/min, the column temperature at 30 °C, and the total run time at 10.5 min. The injection volume was 10 μL and mass spectrometric conditions were established as follows: ionization mode positive (electrospray), acquisition mode multiple reaction monitoring (MRM), gas flow rate of 5 L/min, ion source temperature of 300 °C, sheath gas temperature of 400 °C, nebulizer pressure set at 3500 V, and nozzle voltage at 500 V. Nitrogen was employed as both the nebulizing and collision gas.

Data acquisition and quantitative analysis were performed using MassHunter software (Agilent, version 10.1). The precursor ion, product ions, and optimized collision energies for AA and the internal standard AA-d3 are presented in Table 4.

### 3.5. Statistical Analysis

All experiments were performed in triplicate (*n* = 3). All statistical analyses were performed using SPSS 23 statistical software (IBM Corp., Armonk, NY, USA). Differences in the variations in AA concentrations between the formula groups according to the main ingredient were analyzed using one-way analysis of variance (ANOVA), and the significance of the differences between groups, excluding the group with a single observation (amino acid-based), was determined using Tukey’s multiple comparison test. Statistically, the lowest level of significance was taken as *p* < 0.05.

## 4. Conclusions

Since AA poses significant health hazards, including carcinogenicity and neurotoxicity, the application of effective mitigation and analytical strategies is essential to protect consumers and comply with international safety standards. AA represents a particularly high health risk for infants due to their greater daily dietary intake per kilogram of body weight, higher resting metabolic and respiratory rates, and larger body surface area compared with adults.

In this study, cereal-based formulas -particularly those containing added glucose syrup- showed the highest mean AA concentration (188.1 ± 100.8 µg/kg) and significantly higher than goat’s milk-based (52.7 ± 25.67), plant-based (48.8 ± 31.68), and cow’s milk-based (27.5 ± 29.62) formulas (*p* < 0.001). However, an amino acid-based sample intended for infants under one year of age exhibited the single highest AA value among all analyzed samples, this sample also contained glucose syrup. Conversely, the cow’s milk-based formulas of the same brand (B1) formulated with glucose syrup exhibited significantly lower AA levels. These findings indicate that differences in formulation, ingredient composition, and processing conditions play a crucial role in determining AA formation in the final product.

Given the potential health risks of AA exposure during infancy and the complexity of the infant formula matrix, regular monitoring using highly selective analytical methods with efficient extraction and clean-up steps is vital to ensure product safety. Although chromatographic techniques offer excellent sensitivity and detection capability for AA analysis, their widespread implementation remains limited due to matrix complexity, high operating costs, time demands, and environmental considerations.

In this context, a new extraction and clean-up protocol was developed to obtain clear extracts for the reliable determination of AA in infant formulas by LC-QqQ-MS. The method integrates sequential hydration with water, protein precipitation using ACN, and dual-sorbent clean-up employing PSA and C18 materials. The validated methodology effectively minimized matrix effects, improved extract clarity, and ensured high reproducibility, outperforming previously examined protocols. Overall, this approach provides a robust and reliable tool for the routine analysis of AA in infant formulas, contributing to improved food safety and quality assurance.

## Figures and Tables

**Table 1:** Summary of infant formula samples (*n* = 31) grouped by age category, main ingredient, and formula type.

Age Group	Main Ingredient	Formula Type	Sample (Brand Code)
0–6 months	Cow’s milk-based	Standard formula	9 (B1, B10, B2, B3, B7, B8)
	Cow’s milk-based	Therapeutic/Special	2 (B2, B4)
	Goat’s milk-based	Standard formula	3 (B12, B6, B9)
6–12 months	Cow’s milk-based	Standard formula	1 (B2)
<1 year	Cow’s milk-based	Therapeutic/Special	5 (B1)
	Plant-based	Therapeutic/Special	2 (B1, B2)
	Amino-acid based	Therapeutic/Special	1 (B1)
>6 months	Cereal-based	Standard formula	1 (B1)
	Cow’s milk-based	Therapeutic/Special	1 (B4)
	Goat’s milk-based	Standard formula	1 (B12)
>1 year	Cow’s milk-based	Standard formula	2 (B2, B5)
	Goat’s milk-based	Standard formula	1 (B11)
0–36 months	Cereal-based	Therapeutic/Special	1 (B4)
	Cow’s milk-based	Therapeutic/Special	1 (B2)

**Table 2:** AA concentrations in infant formulas by age group, main ingredient, and brand (μg/kg).

Age Group	Main Ingredient	Brand Code	n	Mean	Min	Max
0–6 months	Cow’s milk-based	B1	2	11.5	<LOQ	23.1
		B2	4	19.0	<LOQ	76.0
		B3	1	74.4	74.4	74.4
		B4	1	77.5	77.5	77.5
		B7	1	25.8	25.8	25.8
		B8	1	24.8	24.8	24.8
		B10	1	76.1	76.1	76.1
	Goat’s milk-based	B6	1	66.1	66.1	66.1
		B9	1	26.8	26.8	26.8
		B12	1	66.8	66.8	66.8
	Mean		14	38.4	<LOQ	77.5
6–12 months	Cow’s milk-based	B2	1	23.4	23.4	23.4
<1 year	Cow’s milk-based	B1	5	25.7	<LOQ	71.8
	Plant-based	B1	1	26.4	26.4	26.4
		B2	1	71.2	71.2	71.2
	Amino acid-based	B1	1	268.2	268.2	268.2
	Mean		8	61.8	<LOQ	268.2
>6 months	Cereal-based	B1	1	259.4	259.4	259.4
	Cow’s milk-based	B4	1	24.0	24.0	24.0
	Goat’s milk-based	B12	1	80.0	80.0	80.0
	Mean		3	121.1	24.0	259.4
>1 year	Cow’s milk-based	B2	1	0.0	<LOQ	0.0
		B5	1	23.3	23.3	23.3
	Goat’s milk-based	B11	1	23.7	23.7	23.7
	Mean		3	15.7	<LOQ	23.7
0–36 months	Cereal-based	B4	1	116.8	116.8	116.8
	Cow’s milk-based	B2	1	0.0	<LOQ	0.0
	Mean		2	58.4	<LOQ	116.8

“n” represents the number of individual samples analyzed for each brand code, corresponding to distinct product or batches. Each sample was analyzed by triplicate.

**Table 4:** LC-MS parameters for AA analysis.

Compound	Precursor Ion (*m*/*z*)	Adduct	Fragmentor Voltage (V)	Product ion (*m*/*z*) †	Collision Energy (eV)	Retention Time (min)
**AA**	72.2	[M + H]^+^	40	**55.1**	10	6.3
				44.2	15	
				27.1	15	
**AA-d3**	75.2	[M + H]^+^	40	**58.2**	10	6.3
				44.2	25	
				30.1	15	

† Quantifier ion in bold; Abbreviations: AA: Acrylamide.

## Data Availability

Dataset available on request from the authors.

## Supplementary Materials

The following supporting information can be downloaded at: https://www.mdpi.com/article/10.3390/molecules30244718/s1, Table S1: Description of the infant formula samples analyzed in this study, including age category, formula type based on main ingredient, brand code, primary protein and carbohydrate source, and AA level detected (μg/kg); Table S2: AA concentrations in infant formulas by main ingredient (μg/kg).

## Abbreviations

The following abbreviations are used in this manuscript:

AA	Acrylamide
ACN	Acetonitrile
d-LLME	Dispersive liquid liquid microextraction
d-SPE	Dispersive solid phase extraction
GC	Gas chromatographic
GC-MS	Gas chromatographic-mass spectrometry
HPLC	High-performance liquid chromatography
HLB	Hydrophilic–lipophilic balanced
HBr	Hydrobromic acid
IARC	International Agency for Research on Cancer
ID-LC/MS	Isotope dilution–liquid chromatography/mass spectrometry
K_2_HPO_4_	Dipotassium hydrogen phosphate
KOH	Potassium hydroxide
LC	Liquid chromatography
LC-QqQ-MS	Liquid chromatography triple quadrupole mass spectrometry
LC-MS	Liquid chromatography-mass spectrometry
LOD	Limit of detection
LOQ	Limit of quantification
ME	Matrix effect
MeOH	Methanol
MgSO_4_	Magnesium sulfate
MS	Mass spectrometric
MSPD	Matrix solid-phase dispersion
MCX	Mixed-mode cation exchange
NaCl	Sodium chloride
PSA	Primary secondary amine
PVDF	Polyvinylidene fluoride
RSDs	Relative standard deviations
SLE	Solid liquid extraction
SPE	Solid phase extraction
UPLC	Ultra performance liquid chromatography
TQ	Triple quadrupole

## References

[1] WHO (2017). Guidance on Ending the Inappropriate Promotion of Foods for Infants and Young Children: Implementation Manual. https://www.who.int/publications/i/item/9789241513470.

[2] Bakshi S., Paswan V.K., Yadav S.P., Bhinchhar B.K., Kharkwal S., Rose H., Kanetkar P., Kumar V., Al-Zamani Z.A.S., Bunkar D.S. (2023). A comprehensive review on infant formula: Nutritional and functional constituents, recent trends in processing and its impact on infants’ gut microbiota. Front. Nutr..

[3] Boyaci-Gunduz C.P. (2022). Acrylamide exposure of infants and toddlers through baby foods and current progress on regulations. Curr. Opin. Food Sci..

[4] Jiang S.L., Guo M.R., Guo M. (2021). 8–Processing technology for infant formula. Human Milk Biochemistry and Infant Formula Manufacturing Technology.

[5] Varghese K.S., Pandey M.C., Radhakrishna K., Bawa A.S. (2014). Technology, applications and modelling of ohmic heating: A review. J. Food Sci. Technol..

[6] Sun X., Wang C., Wang H., Guo M. (2018). Effects of Processing on Structure and Thermal Properties of Powdered Preterm Infant Formula. J. Food Sci..

[7] Aktağ I.G., Hamzalıoğlu A., Kocadağlı T., Gökmen V. (2022). Dietary exposure to acrylamide: A critical appraisal on the conversion of disregarded intermediates into acrylamide and possible reactions during digestion. CRFS.

[8] IARC (1994). Acrylamide. Monographs on the Evaluation of Carcinogenic Risks to Humans: Some Industrial Chemicals.

[9] Lupăescu A.-V., Oroian M. (2025). Advancements and obstacles in acrylamide detection and mitigation in food products. Food Chem. X.

[10] Başaran B., BÇuvalcı B., Kaban G. (2023). Dietary Acrylamide Exposure and Cancer Risk: A Systematic Approach to Human Epidemiological Studies. Foods.

[11] Abd Al Haleem E.N., Hasan W.Y.S., Arafa H.M.M. (2022). Therapeutic effects of thymoquinone or capsaicin on acrylamide-induced reproductive toxicity in rats mediated by their effect on oxidative stress, inflammation, and tight junction integrity. Drug Chem. Toxicol..

[12] Üremïş N., Üremïş M.M., Gül M., Özsoy E.N., Türköz Y. (2024). Protective effects of vitamin E against acrylamide-induced hepatotoxicity and nephrotoxicity from fetal development to adulthood: Insights into Akt/NF-κB and Bcl-xL/Bax signaling pathways. Toxicology.

[13] (2019). IARC. Report of the Advisory Group to Recommend Priorities for the IARC Monographs During 2020–2024. Lyon, France. https://monographs.iarc.who.int/wp-content/uploads/2019/10/IARCMonographs-AGReport-Priorities_2020-2024.pdf.

[14] EFSA (2015). EFSA Panel on Contaminants in the Food Chain, Scientific opinion on acrylamide in food. Efsa J..

[15] Mousavi-Khaneghah A., Fakhri Y., Nematollahi A., Seilani F., Vasseghian Y. (2022). The Concentration of Acrylamide in Different Food Products: A Global Systematic Review, Meta-Analysis, and Meta-Regression. Food Rev. Int..

[16] Palus K. (2024). Dietary Exposure to Acrylamide Has Negative Effects on the Gastrointestinal Tract: A Review. Nutrients.

[17] Ghiasi R., Mohammadi A., Kamankesh M., Barzegar F., Jazaeri S. (2022). Risk Evaluation of Acrylamide in Powder Infant Formula Based on Ingredient and Formulation in Three Critical Age Groups of Children Below 2 Years Old: Efficient Microextraction Followed by GC–MS Analysis Based on CCD. Food Anal. Methods.

[18] López-Ruiz R., Marin-Sáez J., Romero-González R., Garrido-Frenich A. (2025). Impact of pectin addition on acrylamide formation in bakery products: Mitigation strategies. J. Sci. Food Agric..

[19] López-Ruiz R., Marin-Saez J., Cunha S.C., Fernandes A., de Freitas V., Viegas O., Ferreira I.M. (2023). Fibre enrichment of cookies to mitigate acrylamide formation and gastrointestinal bioaccessibility. LWT.

[20] European Commission (2022). Analytical Quality Control and Method Validation Procedures for Pesticide Residues Analysis in Food and Feed. Supersedes Document No. SANTE/11312/2021. https://www.eurl-pesticides.eu/docs/public/tmplt_article.asp?CntID=727.

[21] Pacetti D., Gil E., Frega N.G., Álvarez L., Dueñas P., Garzón A., Lucci P. (2015). Acrylamide levels in selected Colombian foods. Food Addit. Contam. Part B.

[22] Lambert M., Inthavong C., Hommet F., Leblanc J.C., Hulin M., Guerin T. (2018). Levels of acrylamide in foods included in ‘the first French total diet study on infants and toddlers’. Food Chem..

[23] Başaran B., Aydın F. (2022). Determination of acrylamide levels in infant formulas and baby biscuits sold in Turkey. Lett. Appl. NanoBioScience.

[24] Roszko M.Ł., Szczepańska M., Szymczyk K., Rzepkowska M. (2020). Dietary risk evaluation of acrylamide intake with bread in Poland, determined by two comparable cleanup procedures. Food Addit. Contam. Part B.

[25] Pavkovich A.M., Bell D.S., Worsfold P., Poole C., Towsshend A., Miro M. (2019). Extraction | QuEChERS. Encyclopedia of Analytical Science.

[26] Badawy M.E.I., El-Nouby M.A.M., Kimani P.K., Lim L.W., Rabea E.I. (2022). A review of the modern principles and applications of solid-phase extraction techniques in chromatographic analysis. Anal. Sci..

[27] Lee S., Baek S.Y., Han J., Lee J. (2023). Development of a certified reference material for the accurate analysis of the acrylamide content in infant formula. Anal. Bioanal. Chem..

[28] Mastovska K., Lehotay S.J. (2006). Rapid Sample Preparation Method for LC−MS/MS or GC−MS Analysis of Acrylamide in Various Food Matrices. J. Agric. Food Chem..

[29] Esposito F., Nolasco A., Caracciolo F., Velotto S., Montuori P., Romano R., Stasi T., Cirillo T. (2021). Acrylamide in baby foods: A probabilistic exposure assessment. Foods.

[30] Verardo V., Moreno-Trujillo T.R., Caboni M.F., Garcia-Villanova B., Guerra-Hernandez E.J. (2021). Influence of infant cereal formulation on phenolic compounds and formation of Maillard reaction products. J. Food Compos. Anal..

[31] Abt E., Robin L.P., McGrath S., Srinivasan J., DiNovi M., Adachi Y., Chirtel S. (2019). Acrylamide levels and dietary exposure from foods in the United States, an update based on 2011–2015 data. Food Addit. Contam. Part A.

[32] Mojska H.I., Gielecińska Stoś K. (2012). Determination of acrylamide level in commercial baby foods and an assessment of infant dietary exposure. Food Chem. Toxicol..

