# Supplementary material for: Acrylamide Determination in Infant Formulas: A New Extraction Method
Source: Molecules. 2025 Dec 9;30(24):4718. doi: 10.3390/molecules30244718 (PMC12736327; doi:10.3390/molecules30244718)
Supplement: Supplementary file 1 [file molecules-30-04718-s001.zip › molecules-3974165-supplementary.pdf]

Table S1. Description of the infant formula samples analyzed in this study, including age category, formula type based on main ingredient, brand code, primary protein and carbohydrate source, and AA level detected (μg/kg).

Sample number	Brand Code	Age	Main Ingredient	Standard/Therapeutic	Protein and Carbohydrate source	AA (μg/kg) (Mean ± standard deviation)
1	B1	0-6 months	Cow's milk-based	Standard formula	milk protein, lactose	23.1 ± 0.36
2	B1	0-6 months	Cow's milk-based	Standard formula	milk protein, lactose	<LOQ
3	B2	0-6 months	Cow's milk-based	Standard formula	milk protein, lactose	<LOQ
4	B2	0-6 months	Cow's milk-based	Therapeutic/Special	milk protein, lactose	<LOQ
5	B2	0-6 months	Cow's milk-based	Standard formula	milk protein, lactose	76 ± 0.46
6	B2	0-6 months	Cow's milk-based	Standard formula	milk protein, lactose	<LOQ
7	B3	0-6 months	Cow's milk-based	Standard formula	milk protein, lactose	74.4 ± 0.48
8	B4	0-6 months	Cow's milk-based	Therapeutic/Special	milk protein, lactose	77.5 ± 0.34
9	B7	0-6 months	Cow's milk-based	Standard formula	milk protein, glucose syrup	25.8 ± 1.28
10	B8	0-6 months	Cow's milk-based	Standard formula	milk protein, lactose	24.8 ± 0.00
11	B10	0-6 months	Cow's milk-based	Standard formula	milk protein, lactose	76.1 ± 0.87
12	B6	0-6 months	Goat's milk-based	Standard formula	goat milk, lactose	66.1 ± 0.00
13	B9	0-6 months	Goat's milk-based	Standard formula	goat milk, lactose	26.8 ± 0.34
14	B12	0-6 months	Goat's milk-based	Standard formula	goat milk, lactose	66.8 ± 0.44
15	B2	6-12 months	Cow's milk-based	Standard formula	milk protein, lactose	23.4 ± 0.11
16	B1	<1 year	Cow's milk-based	Therapeutic/Special	milk protein, maltodextrin, FOS, GOS	23.8 ± 0.59
17	B1	<1 year	Cow's milk-based	Therapeutic/Special	milk protein, glucose syrup	<LOQ
18	B1	<1 year	Cow's milk-based	Therapeutic/Special	milk protein, lactose	71.8 ± 0.53
19	B1	<1 year	Cow's milk-based	Therapeutic/Special	milk protein, lactose	32.8 ± 0.83

20	B1	<1 year	Cow's milk-based	Therapeutic/Special	milk protein, lactose	<LOQ
21	B1	<1 year	Amino acid-based	Therapeutic/Special	free AA, glucose syrup	268.2 ± 31.19
22	B1	<1 year	Plant-based	Therapeutic/Special	soybean protein, maltodextrin	26.4 ± 2.34
23	B2	<1 year	Plant-based	Therapeutic/Special	soybean protein, maltodextrin	71.2 ± 0.83
24	B1	>6 months	Cereal-based	Standard formula	porridge (oatmeal), free AA, glucose syrup	259.4 ± 24.83
25	B4	>6 months	Cow's milk-based	Therapeutic/Special	milk protein, lactose	24 ± 0.33
26	B12	>6 months	Goat's milk-based	Standard formula	goat milk, lactose	80 ± 3.29
27	B2	>1 year	Cow's milk-based	Standard formula	milk protein, lactose	<LOQ
28	B5	>1 year	Cow's milk-based	Standard formula	milk protein, maltodextrin	23.3 ± 0.62
29	B11	>1 year	Goat's milk-based	Standard formula	goat milk, lactose	23.7 ± 0.42
30	B4	0-36 months	Cereal-based	Therapeutic/Special	rice protein, maltodextrin	116.8 ± 3.12
31	B2	0-36 months	Cow's milk-based	Therapeutic/Special	milk protein, maltodextrin	<LOQ

Table S2. AA concentrations in infant formulas by main ingredient (μg/kg)

Main ingredient	n	Mean ± standard deviation	Minimum	Maximum
Cow's milk-based	21	27.5 ± 29.62[a]	0.0	77.5
Goat's milk-based	5	52.7 ± 25.67[a]	23.7	80.0
Cereal-based	2	188.1 ± 100.83[b]	116.8	259.4
Plant-based	2	48.8 ± 31.68[a]	26.4	71.2
Amino acid-based[†]	1	268.2	268.2	268.2
Total	31	51.0 ± 65.26	0.0	268.2
p[‡]		**< 0.000**		

n, sample size

Note: Values are presented as mean ± SD

[‡]*One-way ANOVA, p was calculated by ANOVA, followed by Tukey's multiple comparison tests excluding the group with a single observation (amino acid-based) (p < 0,05).*

[ab] *different lower letters mean statistically significant difference among the groups (cow's milk-based, goat's milk-based, cereal-based and plant-based) (p < 0.05).*
